# Phosphorylation of RAF Kinase Dimers Drives Conformational Changes that Facilitate Transactivation

**DOI:** 10.1002/anie.201509272

**Published:** 2015-12-08

**Authors:** Pablo G. Jambrina, Nora Rauch, Ruth Pilkington, Katja Rybakova, Lan K. Nguyen, Boris N. Kholodenko, Nicolae‐Viorel Buchete, Walter Kolch, Edina Rosta

**Affiliations:** ^1^ Department of ChemistryKing's College LondonLondonSE1 1DBUK; ^2^ Systems Biology Ireland and Conway InstituteUniversity College Dublin, BelfieldDublin4Ireland; ^3^ Systems Biology IrelandUniversity College Dublin, BelfieldDublin4Ireland; ^4^ Department of Biochemistry and Molecular Biology, Biomedicine Discovery InstituteMonash UniversityMelbourneVictoria3800Australia; ^5^ School of Physics and Complex and Adaptative Systems LaboratoryUniversity College Dublin, BelfieldDublin4Ireland; ^6^ School of Medicine & Medical SciencesUniversity College Dublin, BelfieldDublin4Ireland

**Keywords:** kinases, molecular dynamics, oncogenic signaling, phosphorylation, RAF kinase

## Abstract

RAF kinases are key players in the MAPK signaling pathway and are important targets for personalized cancer therapy. RAF dimerization is part of the physiological activation mechanism, together with phosphorylation, and is known to convey resistance to RAF inhibitors. Herein, molecular dynamics simulations are used to show that phosphorylation of a key N‐terminal acidic (NtA) motif facilitates RAF dimerization by introducing several interprotomer salt bridges between the αC‐helix and charged residues upstream of the NtA motif. Additionally, we show that the R‐spine of RAF interacts with a conserved Trp residue in the vicinity of the NtA motif, connecting the active sites of two protomers and thereby modulating the cooperative interactions in the RAF dimer. Our findings provide a first structure‐based mechanism for the auto‐transactivation of RAF and could be generally applicable to other kinases, opening new pathways for overcoming dimerization‐related drug resistance.

RAF kinases connect the Ras GTPase to activation of the MEK‐ERK pathway. This pathway regulates many fundamental cellular functions, including cell proliferation, and is dysregulated in approximately 50 % of human cancers.^1^ This pathway has thus been a key focus in cancer drug development. A recent breakthrough came with the BRAF inhibitor vemurafenib, which achieved high response rates in BRAF‐mutated metastatic melanoma.^2^ Interestingly, BRAF inhibition in RAS‐mutated tumors induces paradoxical ERK activation and tumor progression owing to the formation of RAF dimers.^3^ RAF dimerization is also a major mechanism of acquired clinical resistance to RAF inhibitors.^4^ Owing to its important clinical implications, RAF dimerization has attracted enormous interest. RAF homo‐ and heterodimers show significantly higher kinase activity than monomers, and it has been shown that physiological RAF activation involves dimerization.^1^, ^5^ Dimer activity remains high even when one protomer (denoted as the activator) is kinase‐dead or inhibited, owing to allosteric transactivation of its binding partner (the receiver).^1^, ^5^ Recent data indicate that the N‐terminal acidic (NtA) motif^6^ is essential for the allosteric activation of RAF dimers.^7^ This region is located just upstream of the kinase domain and mediates physiological activation. In RAF1, phosphorylation of the corresponding sequence SSYY (residues 338–341) is induced during RAF1 activation.6b, 8 In BRAF (residues 446–449, sequence SSDD), the activating site S446 is constitutively phosphorylated and the tyrosines are replaced by negatively charged aspartates. This configuration of the NtA motif primes BRAF for activation, which may explain why single mutations of BRAF, such as V600E in the activation loop, can cause full activation and drive cancer, while RAF1 mutations are rare in cancer.^9^ There is no consensus on which kinase phosphorylates the NtA motif in vivo, since several kinases, including RAF1 itself, have been reported to be able to do this.^1^, ^10^ As shown by mutagenesis studies,^7^ the NtA motif in the activator is required for transactivation of the receiver in RAF dimers. However, no structural evidence is available to explain this allosteric activation process, since all RAF crystal structures lack the NtA motif.

Based on recently available crystallographic data for RAF (e.g., PDB entries 4E26^11^ and 3OMV3b) we modeled RAF homo‐ and heterodimers that include the NtA region and correspond to the smallest subset of residues present at the N terminus of the kinase domain in constitutively dimerized, drug resistant splice variants^4^ (Figure 1 a). By using atomistic molecular dynamics (MD) simulations of kinase dimers,^12^ we also investigated the role of phosphorylation, which is biochemically well documented but has eluded detailed structural studies. The computational modeling approach and the parameters used are described in the Methods section of the Supporting Information and summarized in Table S1.

**Figure 1:**
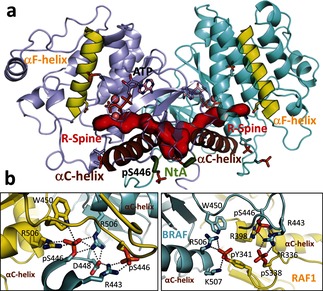
a) Structure of the phosphorylated BRAF homodimer with bound ATP (ATP‐^P^BRAF), obtained from MD simulations. Key structural elements are highlighted: the NtA motif, the R‐spine, and the αC and αF helices. b) Interactions of the NtA motif within the phosphorylated BRAF homodimer and the phosphorylated BRAF–RAF1 heterodimer. The newly formed interchain ion pairs are highlighted for protomers A and B (cyan and yellow, respectively). See also Figures S1–S3.

Phosphorylation of the NtA motif generates several salt bridges that extend and stabilize the binding interface between two BRAF protomers (Figure 1 b). Intriguingly, these salt bridges are primarily interprotomer salt bridges, which are formed between the NtA motif and positive residues located either upstream of the NtA motif or at the C‐terminal end of the αC‐helix, the orientation of which plays an important role in kinase activation.12a, 13

We note that all of the residues that form interprotomer salt bridges with phosphorylated residues of the NtA motif are conserved in the three RAF isoforms, but not in other kinases (Table S2 in the Supporting Information). Remarkably, the C‐terminal end of the αC‐helix is neutral in most kinases, whereas in RAFs it carries a +3 charge. Moreover, mutations of these charged residues impair kinase activity^14^ and affect RAF homo‐ and heterodimerization.3b, 14, 15 Since RAF kinases dimerize via the C‐terminal end of the αC‐helix, the accumulation of six positive charges at the dimerization interface enables interaction with the highly negatively charged NtA motif (specific to RAF), thereby promoting the dimerization. In fact, we estimate that NtA phosphorylation constitutes almost half of the interaction potential energy between the BRAF protomers (Figure 2 a and Table S1), predominantly by enhancing electrostatic interactions. In particular, we identified two conserved Arg residues, R443 (R336) at the NtA region and R506 (R398) close to the αC‐helix in BRAF (RAF1), that participate in the intermolecular salt bridges. R506 (R398) has been reported previously to play a role in dimerization,^14^ and here we confirmed the relevance of R443 (R336) experimentally by co‐immunoprecipitation, demonstrating a reduced dimerization propensity of R→A mutants (Figure 2 b). The differences between BRAF and RAF1 in terms of their cellular phosphorylation states (i.e., BRAF is constitutively phosphorylated whereas RAF1 activation requires additional input6b) and the exact sequences of the NtA motifs (Figure 1 b, and Figures S1–S3 in the Supporting Information) explain the significant differences between the activities of BRAF and RAF1 homo‐ and heterodimers.5c

**Figure 2:**
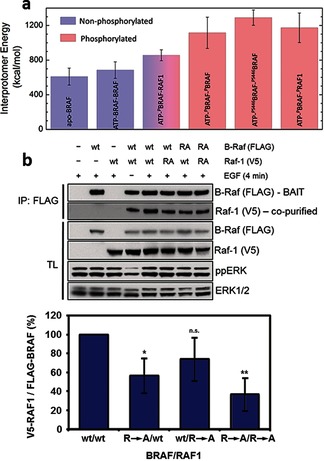
a) Average interaction potential energy (see the Supporting Information for details) between protomer A and protomer B for BRAF homo‐ and heterodimers. Only the last 75 % segment of the simulation was considered. The results for the phosphorylated dimers include contributions for three different trajectories. For the phosphorylated BRAF homodimers, we show results where i) all the activating regulatory residues (S446, S579, T599 and S602) are phosphorylated (pBRAF), and ii) only the NtA motif was phosphorylated (pS446 BRAF). b) BRAF R443A and RAF1 R336A mutants show reduced heterodimerization. Mutants of BRAF and RAF1 were transiently overexpressed in HEK293T cells. Dimerization was analyzed by co‐immunoprecipitation (IP) from EGF‐stimulated cells. V5=V5‐tag, FLAG=FLAG‐tag, TL=total lysate, *p=0.0192, **p=0.0058, n.s.=not significant (p=0.1108).

Our simulations also reveal that the phosphorylated NtA motif is connected to the active site via the R‐spine^16^ (Figure 3 a). This conserved hydrophobic structure connects four residues from critical sites in the kinase monomer,16a, 17 including the active site. The R‐spine is anchored to the αF‐helix via a hydrogen bond between a carboxylate group at the N‐terminal end of the αF‐helix and the backbone of the HRD motif.16b An assembled R‐spine indicates active kinase conformations, while a broken R‐spine correlates with inactive kinase conformations.^16^

**Figure 3:**
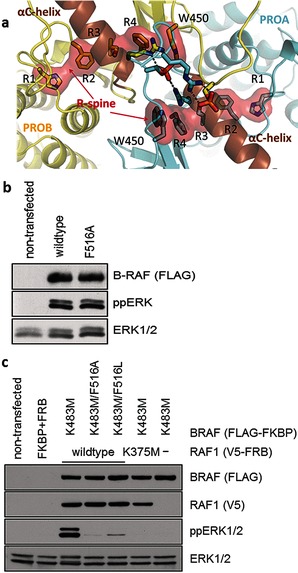
a) R‐spine of the two protomers of ATP‐^P^BRAF. R1–R4 denotes the four residues that form the R‐spine (H574, F595, L505, and F516 for BRAF). The conserved Trp (W450 for BRAF) extends the R‐spine, connecting it with the dimerization interface. b) F516A mutation does not affect the activity of BRAF. HEK293T cells were transfected with FLAG‐tagged BRAF mutants (as indicated). Lysates were collected the next day from growing cells. c) F516 is required for transactivation activity of kinase‐dead BRAF. HEK293T cells were transiently transfected with FLAG‐FKBP‐tagged BRAF and V5‐FRB‐tagged RAF1. Heterodimer formation in serum‐starved cells was induced by addition of 500 nm A/C heterodimerizer for 1 hour. Total lysates were collected and analyzed. Substitution of F516 in kinase‐dead BRAF K483M resulted in reduced ERK activation by RAF heterodimers.

Previous experiments showed that mutation of a conserved Trp residue (W450 for BRAF, Table S2) impedes transactivation and impairs the dimerization of BRAF.^7^ We observed that W450 extends the R‐spine by forming stacking interactions with the fourth residue (R4, Figure 3 a). Although W450 is highly conserved in most kinases (Table S2), the interaction between this Trp and the R4 residue of the R‐Spine is not always observed. R4 is aliphatic in about 80 % of the kinases, including PKA. In PKA, CDK2, and p38 structures, the R‐spine and the corresponding Trp do not interact. Although this Trp is not conserved in EGFR, crystal structures show that its functional equivalent lysine (L680)^18^ also interacts with the R‐spine. Remarkably, mutation of L680 destabilizes the EGFR dimer in the active complex and impedes kinase activity.^18^ We observed that the NtA motif together with W450 extends the dimerization interface, thereby connecting the R‐spines and thus the distant active sites of the two protomers. For PKA, aliphatic‐to‐aromatic mutation of R4 gives normal levels of catalytic activity.^19^ This is also the case for BRAF F516A and F516L mutations, which maintain the catalytic activity (Figure 3 b) because they preserve the integrity of the R‐spine within the monomer. However, we found that these BRAF mutants are unable to transactivate RAF1 in the heterodimer (Figure 3 c). These results provide a first structure‐based explanation for the transactivation mechanism of RAF dimers following NtA motif phosphorylation.

Importantly, our results indicate phosphorylation‐induced large‐scale structural changes in RAF dimers, whereas the unphosphorylated dimers remain structurally similar to their starting structures, in agreement with the crystallographic structures. Only when all phosphorylated residues, including the two activation‐loop residues near V600, are present in the simulated system, we observe large changes between the universally conserved HRD and DFG motifs (Figure 4). These changes provide insight into the structural flexibility present in the kinase domains, and could provide a structural explanation for recent observations on transactivation.^7^ Remarkably, these changes involve structural elements that are connected via the R‐spine and/or the NtA motif, and they typically occur in only one of the protomers, thereby giving rise to asymmetry in the dimer. Interestingly, only structures that include vemurafenib or closely related inhibitors (PDB entries 3OG7,^20^ 4FK3,^21^ and 3C4C^21^) present asymmetrical dimers, which have been proposed as a general mechanism for the allosteric modulation of kinase activity.^22^ Our protomer structures differ from these inactive crystal structures, since the presence of ATP enhances the stability of the salt bridge between K483 and E501, which is absent in the vemurafenib‐bound structures. Therefore, our protomer structures could be useful leads for the development of drugs that avoid the paradoxical kinase activation resulting from drug‐induced RAF dimerization.

**Figure 4:**
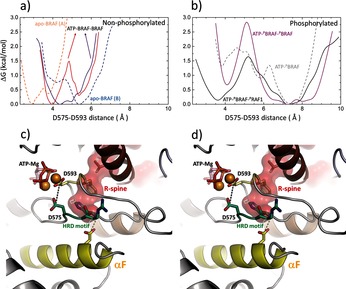
a) Free energies along the D575–D593 distance. a) Simulations M1 and M2 (without phosphorylated residues). b) Simulations M3a, M5a, and M8 (with phosphorylation taken into account). c, d) Snapshots of the MD simulation structures at the beginning (c), and at the end (d) of the simulation trajectory M3a, highlighting the D575–D593 distance and the corresponding detachment of the R‐spine from the anchoring αF‐helix.

In summary, our results show that phosphorylation of the NtA motif promotes dimerization through several interprotomer salt bridges formed between the NtA motif of one of the protomers and the positively charged C‐terminal end of the αC‐helix of the other protomer. They further reveal the importance of the conserved tryptophan residue located at the N‐terminal end of the kinase domain (W450 for BRAF), which plays a crucial role by connecting the R‐spines of the two protomers. This suggests a cooperative interprotomer interaction that is mediated by salt bridges involving the phosphorylated NtA motif. The direct interaction of the R‐spines via W450 explains why mutation of W450 abolishes the transactivation of RAF dimers.^7^ More importantly, this also explains how the phosphorylated dimers undergo significant conformational changes. These changes involve detachment of the R‐spine from the anchoring αF‐helix, and significant changes to the residue pair distances between the HRD and DFG motifs. The asymmetric transactivation mechanism of RAF kinases also provides a structural basis for understanding the paradoxical activation caused by type I and type IIB inhibitors.^23^

## Supporting information

As a service to our authors and readers, this journal provides supporting information supplied by the authors. Such materials are peer reviewed and may be re‐organized for online delivery, but are not copy‐edited or typeset. Technical support issues arising from supporting information (other than missing files) should be addressed to the authors.

Supplementary
