# Supplementary material for: Phosphorylation of RAF Kinase Dimers Drives Conformational Changes that Facilitate Transactivation
Source: Angew Chem Int Ed Engl. 2015 Dec 8;55(3):983–6. doi: 10.1002/anie.201509272 (PMC4736688; doi:10.1002/anie.201509272)
Supplement: Supplementary file 1 — Supplementary [file ANIE-55-983-s001.pdf]

Angewandte Chemie
Eine Zeitschrift der Gesellschaft Deutscher Chemiker

Supporting Information

# Phosphorylation of RAF Kinase Dimers Drives Conformational Changes that Facilitate Transactivation

*Pablo G. Jambrina, Nora Rauch, Ruth Pilkington, Katja Rybakova, Lan K. Nguyen, Boris N. Kholodenko, Nicolae-Viorel Buchete, Walter Kolch,* and Edina Rosta**

anie_201509272_sm_miscellaneous_information.pdf

# Extended methods

## Molecular dynamics simulations

Our initial conformations for the molecular dynamics (MD) simulations were based on the PDB structures 4E26 [1] and 3OMV [2] corresponding to the active forms of the BRAF and RAF1 kinase domain, respectively. The ATP and the two $Mg^{2+}$ ions were docked in the active site based on the 4DFX structure [3]. The initial coordinates of the missing residues (439-447, and 604-609) were modeled using the M4T server [4-6]. Unless otherwise stated, all the homodimer systems included a short, 20 amino acid-long substrate-like peptide (SP20 [3]) bound in the active site of each monomer in the simulations of ATP-BRAF (system M2) and of the ATP-pBRAF (system M3) dimers. This peptide corresponds to a substrate of the protein kinase A (PKA), used in our homology-based model of BRAF (see Methods), and it was kept in our simulations to model BRAF substrates. The initial models were essentially symmetrical, the all-atom RMSD between the protomers was 0.07 Å and the dimer interactions are remarkably similar (all-atom RMSD = 0.7 Å obtained for the alignment of the same dimer but with the positions of the protomers inter-switched). During the writing of this article, a crystal structure of the BRAF-MEK complex was released [7] in which, to prevent the phosphorylation of MEK to happen, MEK was co-crystallized in the presence of a non-competitive ATP inhibitor called G-573. The position of S222 of MEK in the MEK-BRAF complex resembles the position of S21 (of SP20) in our model and, if the BRAF monomers are aligned, they are quite close (less than 8 Å).

**BRAF-BRAF homodimers.** We performed MD simulations of four types of BRAF homodimeric systems: (1) system type 1 (M1), the apo-BRAF homodimer, (2) system type 2 (M2) the ATP-BRAF homodimer with ATP and two $Mg^{2+}$ ions bound in both active sites, (3) system type 3 (M3, Figure 1) the ATP-pBRAF homodimer also with ATP and two $Mg^{2+}$ ions bound in both active sites and with phosphorylated activating residues: S446 in the NtA motif, T599 and S602 in the activation loop, and S579 in the catalytic domain, and (4) system type 4 (M4) the ATP-pBRAF homodimer with the S446 residue phosphorylated and with ATP and two $Mg^{2+}$ ions bound per active site. A single trajectory was generated for M1, M2, and M4 and three trajectories were run for M3 using identical initial conditions.

**BRAF-RAF1 heterodimers.** We performed MD simulations for the BRAF-RAF1 phosphorylated heterodimer (M5) with phosphorylated activating residues in both kinases (BRAF: S446, T599, S602 and S579; RAF1: S338, Y341, S471, T491, and S494), and for the heterodimer where only BRAF was phosphorylated (M6). Three trajectories were run for m5 and one for M6. For these heterodimers, the SP20 peptide was not included.

**RAF1 homodimers**. We also carried out MD simulations for the RAF1-RAF1 phosphorylated homodimer (M7) with phosphorylated activating residues (S338, Y341, S471, T491, and S494), one ATP and two $Mg^{2+}$ per active site.

**BRAF monomers**. Simulations were carried out for BRAF monomers in the absence of ATP and $Mg^{2+}$ in the active site and where all the regulatory residues are phosphorylated (M8).

**Long MD simulations.** We performed long MD simulations for BRAF homodimers, BRAF-RAF1 heterodimers and RAF1 homodimers in explicit water using the CHARMM-27 force field [8, 9], with the simulation lengths between 100 ns and 300 ns (Table S1). All simulations were performed at constant temperature (298 K) and pressure (1 bar). The initial structures were prepared using CHARMM-GUI [10]. Coordinates of the Hydrogen atoms were generated with CHARMM[11, 12] using standard protonation states for all the titrable residues, i.e., the Lys and Arg residues were positively charged and the ATP, Asp and Glu residues were negatively charged. The protein complexes were placed in the center of a cubic TIP3 water box large enough to contain the protein complex and at least 10 Å of solvent on all sides. Additionally, K+ and Cl- ions were added to the simulation systems to account for a 0.10M KCl. All atom MD simulations were run using NAMD [13]. The Particle Mesh Ewald method was used for the electrostatics of the periodic boundary conditions [14]. Energy minimizations were performed by first optimizing the coordinates of the protein sidechains and all water molecules in 9000 steps. Subsequently, all the atomic coordinates were optimized for 20000 steps. Finally, the simulation was equilibrated during 3 ns constraining the coordinates of the non-hydrogen atoms of the protein to its initial position. Langevin dynamics was used with a Langevin damping coefficient of 1 $ps^{-1}$. For long range electrostatics treatment, the non-bonded switching distance was set to 10 Å and a cut off distance of 12 Å was used. The ShakeH algorithm [15] was used with 2 fs time steps. For the subsequent analysis, the coordinates were saved every 0.1 ns.

All the molecular models of the systems studied here present secondary structural elements that remained stable during our atomistic MD simulations. The converged values for the inter-monomer interaction energies, and the dimer and protomer root-mean-square deviation (RMSD) values (i.e., calculated with respect to the initial conformation based on the PDB structure 4E26 [1], and 3OMV [2] including the backbone of the residues that form part of a α-Helix or a β-sheet) are reported in Table S1.

**Interprotomer Energy.** We calculated the interaction energy between protomers A and B throughout the simulation, by computing the non-bonding interactions using the force-field parameters (basically charge-charge interactions). For structural reasons we included a small segment (residues 721-730) just downstream the kinase domain in our initial simulations. As it does not play any significant role, this small segment was not considered for the interaction energy calculations.

## Experimental Procedures

**Cells.** HEK293T cells were grown in DMEM, supplemented with 10% fetal bovine serum and 2mM L-Glutamine (all from Gibco-BRL). Cells were transiently transfected using Lipofectamine 2000 (Invitrogen) or TransIT-X2 (Mirus) according to manufacturer's instructions. Before each experiment, cells were serum starved overnight. If required, cells were stimulated with 10nM Epidermal Growth Factor (hEGF, Roche) for 4 minutes. Heterodimerization of FKBP- and FRB-tagged proteins was induced by addition of 500nM A/C Heterodimerizer (Clontech) for 1 hour.

**Antibodies.** Rabbit anti-phospho-MEK1/2 (#9121), rabbit anti-total MEK1/2 (#9122), and rabbit anti-phospho-ERK1/2 (#9101) were from Cell Signaling. Mouse monoclonal anti-phospho-ERK1/2 (#8159), rabbit polyclonal anti-total ERK1/2 (#5670), and mouse monoclonal anti-Flag(M2)-HRP (#8592) were from Sigma- Aldrich. Mouse monoclonal anti-V5-HRP (R961-25) was from Life Technologies.

**Expression plasmids.** Flag-tagged BRAF was described before [16]. V5-tagged RAF1 was cloned into pcDNA3.1(+) (Life Technologies). For experiments using induced dimerization, plasmids were based on the iDimerize Inducible Heterodimer System (Clontech): BRAF and RAF1 in pcDNA3.1(+) were N-terminally tagged with FLAG-FKBP and V5-FRB, respectively. Site directed mutagenesis was performed using the QuikChange II XL Site-Directed Mutagenesis Kit (Agilent) according to manufacturer's instructions. Mutants made were BRAF R443A, R505A, K483M, F516A, F516L, D638N, and RAF1 R336A and R398A, and selected double mutants thereof.

**Lysates, Immunoprecipitations and Western Blotting.** Total lysates for western blotting and *in vitro* kinase assay were prepared on ice using RIPA buffer (20 mM TrisHCl, pH 7.4, 150 mM NaCl, 1% Triton-X100, 0.5% deoxycholate, 0.1% SDS). For co-immunoprecipitation experiments, cells were lysed on ice in 10mM TRIS pH 7.5, 150mM NaCl, 0.1% NP-40. All lysis buffers were supplemented with COMPLETE protease and PhosSTOP phosphatase inhibitor cocktails (Roche). Immunoprecipitation was done using anti-Flag (M2) affinity gel (A2220) from Sigma-Aldrich. SDS-PAGE electrophoresis and western blotting were performed using the NuPAGE SDS PAGE Gel System (Life Technologies) or the Mini-PROTEAN III System (Bio-Rad).

**Kinase assays.** BRAF kinase activity was measure by *in vitro* kinase assay as previously described [17]. Briefly, beads were washed after immunoprecipitation 2x with RIPA, 3x with kinase assay buffer (50mM Tris, pH 7.5, 75 mM NaCl, 5mM EGTA, 5 mM MgCl2) and subsequently incubated in 30ul assay buffer containing 200ng recombinant MEK1 (Millipore #14-420) and 100uM ATP for 30 minutes at 30 degrees (shaking). pMEK levels were analysed by Western Blot.

**Table SM1. Interaction energies and RMSD values the different systems studied. The numbers in parentheses are the estimated statistical errors (one standard deviation).**

| Simulation | Time (ns) | Energy[a] | Dimer RMSD[b] |
|---|---|---|---|
| M1: apo-BRAF | 130 | -632 (99) | 2.8 (0.2) |
| M2: ATP-BRAF | 103 | -705 (87) | 2.3 (0.2) |
| M3a: ATP-$^{P}$BRAF | 359 | -1148 (137) | 2.8 (0.2) |
| M3b: ATP-$^{P}$BRAF | 176 | -1251 (56) | 6.0 (0.4) |
| M3c: ATP-$^{P}$BRAF | 150 | -875 (103) | 3.3 (0.4) |
| M4: ATP-$^{pS446}$BRAF | 103 | -1215 (114) | 3.9 (0.6) |
| M5a: ATP-$^{P}$BRAF-$^{P}$RAF1 | 126 | -1096 (186) | 3.1 (0.3) |
| M5b: ATP-$^{P}$BRAF-$^{P}$RAF1 | 155 | -1247 (83) | 2.3 (0.3) |
| M5c: ATP-$^{P}$BRAF-$^{P}$RAF1 | 136 | -1259 (120) | 2.8 (0.2) |
| M6: ATP-$^{P}$BRAF-RAF1 | 90 | -920 (92) | 2.7(0.2) |
| M7: ATP-$^{P}$RAF1-$^{P}$RAF1 | 129 | -1905 (106) | 3.2 (0.2) |
| M8: $^{P}$BRAF monomer | 98 | | 3.3 (0.1) |

[a] Average interaction potential energy between protomer A and protomer B (kcal/mol). Only the last 75% of the simulation has been considered.

[b] Average RMSD between the dimer system and the initial conformation during the simulation.

**Table SM2: NtA-region sequence for three RAF isoforms and five other kinases. The regulatory phosphorylated residues are highlighted in orange, positively charged residues in blue, and negatively charged residues in red. Conserved residues are shaded in gray. The C terminal end of the αC helix is also shown. The alignment with five additional kinases (its sustrate MEK1, EGFR, PKA, LCK, and p38) is also shown. A conserved tryptohan at the beginning of the kinase domain (W450 for BRAF) is highlighted in red. For RAF family of kinases this Trp is adjacent to the NtA motif and its mutation impedes transactivation [18].**

| | | | | | | | | | NtA motif | | | | | | | | | | αC- helix | | | | | | | |
|---|---|---|---|---|---|---|---|---|---|---|---|---|---|---|---|---|---|---|---|---|---|---|---|---|---|---|
| **BRAF** | *439* | K | T | L | G | R | R | D | S | S | D | D | W | E | I | P | ... | 503 | G | V | L | R | K | T | R | H |
| **RAF1** | 331 | R | P | R | G | Q | R | D | S | S | Y | Y | W | E | I | E | ... | 395 | Q | V | L | R | K | T | R | H |
| **ARAF** | 292 | K | N | L | G | Y | R | D | S | G | Y | Y | W | E | V | P | ... | 356 | A | V | L | R | K | T | R | H |
| **MEK1** | 51 | E | A | F | L | T | Q | - | K | Q | K | V | G | E | L | K | ... | 116 | Q | V | L | H | E | C | N | S |
| **EGFR** | 670 | P | S | G | E | A | P | N | Q | A | L | L | R | I | L | K | ... | 764 | Y | V | M | A | S | V | D | N |
| **PKA** | 20 | L | A | K | A | K | E | D | F | L | K | K | W | E | S | P | ... | 93 | R | I | L | Q | A | V | N | F |
| **LCK** | 228 | Q | K | P | Q | K | P | W | W | E | D | E | W | E | V | P | ... | 289 | A | N | L | M | K | Q | L | Q |
| **p38** | 7 | T | F | Y | R | Q | E | L | N | K | T | I | W | E | V | P | ... | 72 | R | L | L | K | H | M | K | H |

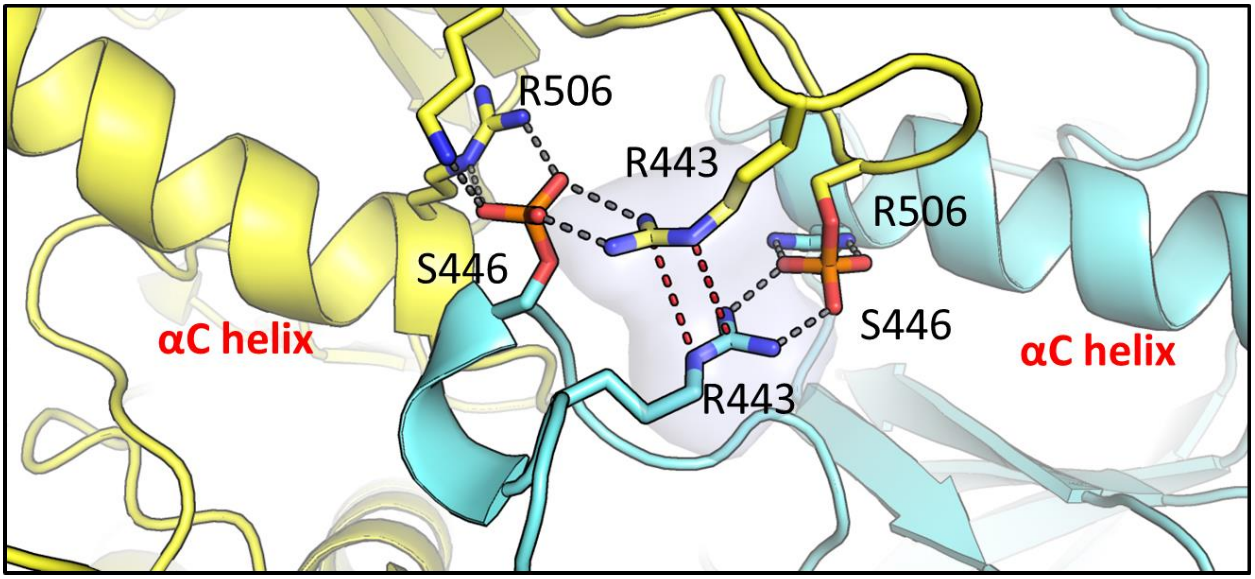


**Figure SM1: Interactions of phosphorylated S446 residues in $^{pS446}$BRAF simulations. The formed interchain ion-pairs are shown for monomers A and B (cyan and yellow, respectively). The π-stacking between both R443 is also highlighted.**

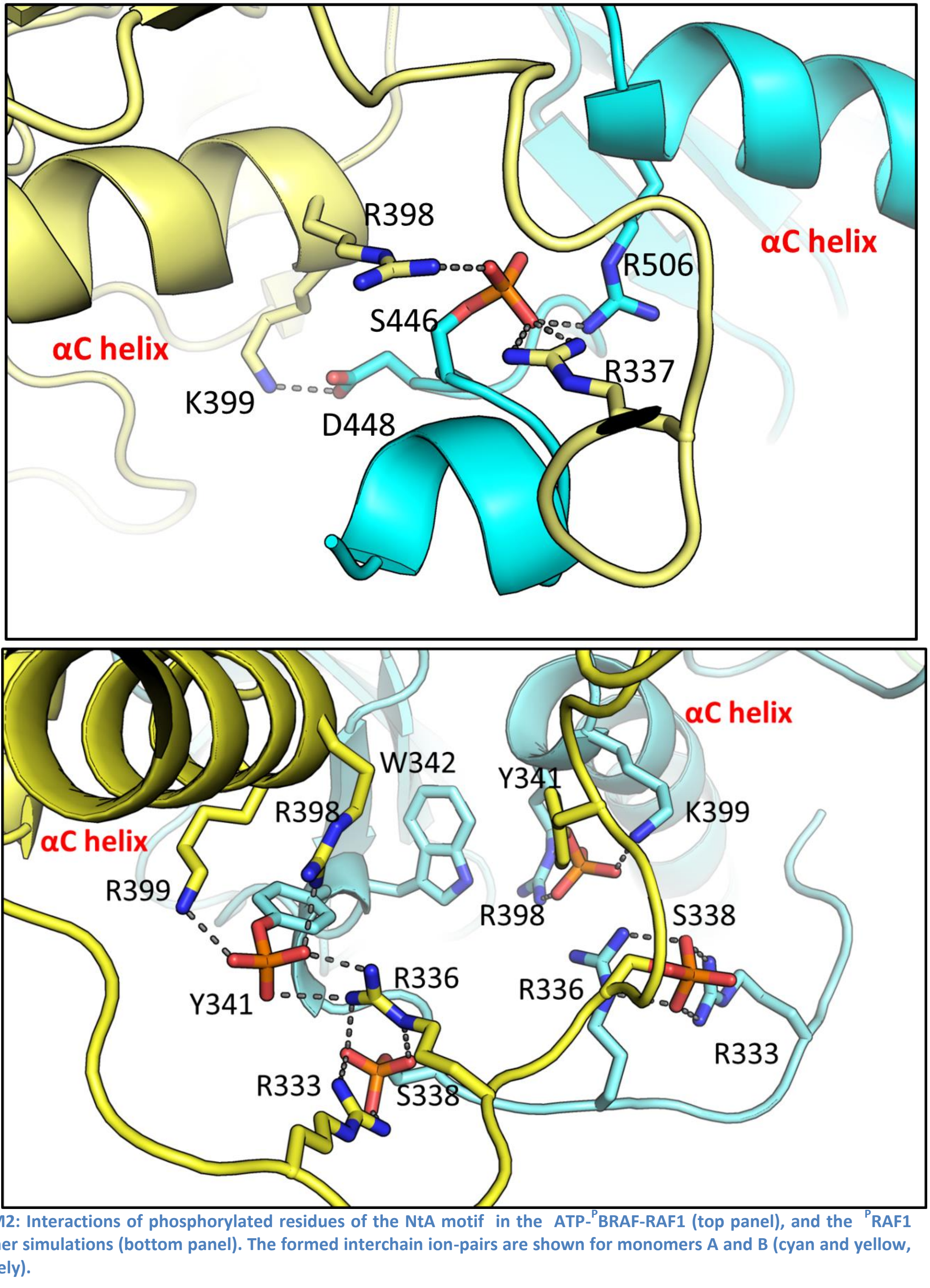


Figure SM2: Interactions of phosphorylated residues of the NtA motif in the ATP-$^{P}$BRAF-RAF1 (top panel), and the $^{P}$RAF1 homodimer simulations (bottom panel). The formed interchain ion-pairs are shown for monomers A and B (cyan and yellow, respectively).

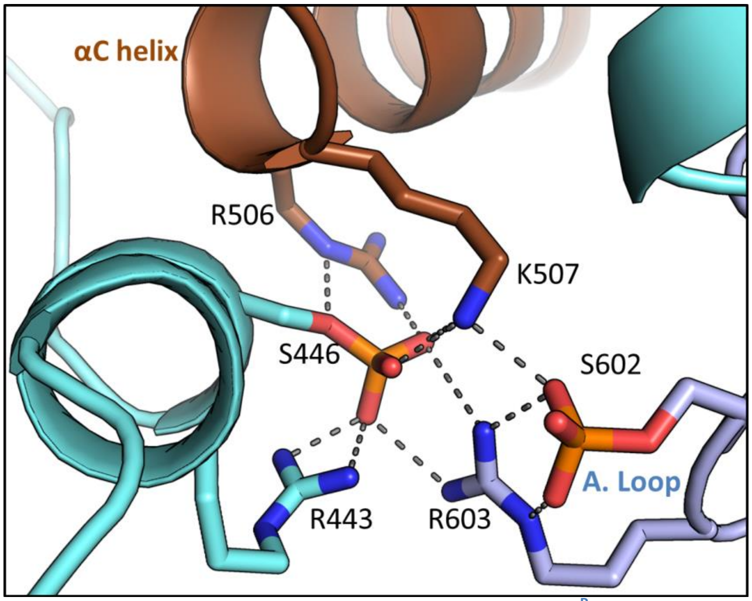


Figure SM3: Interactions of the phosphorylated S446 residue in the $^{P}$BRAF monomer simulation. The ion pairs between pS446 and pS602 are highlighted. The Activation Loop is displayed in slate and the αC helix in brown. For BRAF monomers, the phosphorylated NtA motif is found to create a network of ion-pairs connecting the C-terminal end of the αC-helix with the activation loop and therefore could impair the structural rearrangement of the activation loop necessary for kinase activation. The interaction between K507 and the Activation Loop has been observed in a recent crystal structure of V600E-BRAF monomer [19] and it is thought to be behind the oncogenic activity of the V600E BRAF.

**Free energy calculations.** Free energy profiles were obtained along the D575 and D593 distance. The reaction coordinate was divided into between 20 to 30 states for each simulation, and a transition probability matrix was built from the MD trajectories to determine the corresponding Markov state model, using a lagtime of 0.1 ns [20]. The free energies are calculated from the populations obtained from the eigenvector corresponding to eigenvalue 1.

# References

1. Qin, J., et al., *Identification of a Novel Family of BRAFV600E Inhibitors.* Journal of Medicinal Chemistry, 2012. **55**(11): p. 5220-5230.
2. Hatzivassiliou, G., et al., *RAF inhibitors prime wild-type RAF to activate the MAPK pathway and enhance growth.* Nature, 2010. **464**(7287): p. 431-5.
3. Bastidas, A.C., et al., *Role of N-Terminal Myristylation in the Structure and Regulation of cAMP-Dependent Protein Kinase.* Journal of Molecular Biology, 2012. **422**(2): p. 215-229.
4. Fernandez-Fuentes, N., et al., *M4T: a comparative protein structure modeling server.* Nucleic Acids Res, 2007. **35**(Web Server issue): p. W363-8.
5. Fernandez-Fuentes, N., et al., *Comparative protein structure modeling by combining multiple templates and optimizing sequence-to-structure alignments.* Bioinformatics, 2007. **23**(19): p. 2558-65.

6. Rykunov, D., et al., *Improved scoring function for comparative modeling using the M4T method.* J Struct Funct Genomics, 2009. **10**(1): p. 95-9.
7. Haling, Jacob R., et al., *Structure of the BRAF-MEK Complex Reveals a Kinase Activity Independent Role for BRAF in MAPK Signaling.* Cancer Cell, (0).
8. MacKerell, A.D., et al., *All-Atom Empirical Potential for Molecular Modeling and Dynamics Studies of Proteins†.* The Journal of Physical Chemistry B, 1998. **102**(18): p. 3586-3616.
9. Mackerell, A.D., Jr., M. Feig, and C.L. Brooks, 3rd, *Extending the treatment of backbone energetics in protein force fields: limitations of gas-phase quantum mechanics in reproducing protein conformational distributions in molecular dynamics simulations.* J Comput Chem, 2004. **25**(11): p. 1400-15.
10. Jo, S., et al., *CHARMM-GUI: A web-based graphical user interface for CHARMM.* Journal of Computational Chemistry, 2008. **29**(11): p. 1859-1865.
11. Brooks, B.R., et al., *CHARMM: the biomolecular simulation program.* J Comput Chem, 2009. **30**(10): p. 1545-614.
12. Brooks, B.R., et al., *CHARMM: A program for macromolecular energy, minimization, and dynamics calculations.* Journal of Computational Chemistry, 1983. **4**(2): p. 187-217.
13. Phillips, J.C., et al., *Scalable molecular dynamics with NAMD.* Journal of Computational Chemistry, 2005. **26**(16): p. 1781-1802.
14. Darden, T., D. York, and L. Pedersen, *Particle mesh Ewald: An N·log(N) method for Ewald sums in large systems.* The Journal of Chemical Physics, 1993. **98**(12): p. 10089-10092.
15. Ryckaert, J.-P., G. Ciccotti, and H.J.C. Berendsen, *Numerical integration of the cartesian equations of motion of a system with constraints: molecular dynamics of n-alkanes.* Journal of Computational Physics, 1977. **23**(3): p. 327-341.
16. Gloeckner, C.J., et al., *A novel tandem affinity purification strategy for the efficient isolation and characterisation of native protein complexes.* Proteomics, 2007. **7**(23): p. 4228-34.
17. Hafner, S., et al., *Mechanism of inhibition of Raf-1 by protein kinase A.* Mol Cell Biol, 1994. **14**(10): p. 6696-703.
18. Hu, J., et al., *Allosteric Activation of Functionally Asymmetric RAF Kinase Dimers.* Cell, 2013. **154**(5): p. 1036-1046.
19. Thevakumaran, N., et al., *Crystal structure of a BRAF kinase domain monomer explains basis for allosteric regulation.* Nat Struct Mol Biol, 2015. **22**(1): p. 37-43.
20. Rosta, E. and G. Hummer, *Free Energies from Dynamic Weighted Histogram Analysis Using Unbiased Markov State Model.* Journal of Chemical Theory and Computation, 2015. **11**(1): p. 276-285.
